# Microbial differences between active and remission peri-implantitis

**DOI:** 10.1038/s41598-022-09192-y

**Published:** 2022-03-28

**Authors:** Yuhei Hashimoto, Shinsuke Okada, Keisuke Yasuda, Maiko Kawagoe, Mikihito Kajiya, Kazuhiro Tsuga

**Affiliations:** 1 Department of Advanced Prosthodontics, Graduate School of Biomedical Sciences, Hiroshima University, 1-2-3, Kasumi, Minami-ku, Hiroshima, 734-8553 Japan; 2 Department of Periodontal Medicine, Graduate School of Biomedical Sciences, Hiroshima University, 1-2-3 Kasumi, Minami-ku, Hiroshima, 734-8553 Japan; 3 Division of Dental, Department of Clinical Practice and Support, Hiroshima University Hospital, 1-2-3 Kasumi, Minami-ku, Hiroshima City, 734-8551 Japan

**Keywords:** Diseases, Oral diseases, Peri-implantitis

## Abstract

Peri-implantitis has a polymicrobial etiology and is a major cause of dental implant loss. Various clinical protocols for its prevention and treatment have been proposed; however, some cases show a rapid progression with non-resolving clinical symptoms. To clear a means of differentiating between such cases, the implants with peri-implantitis in this study were categorized as the active group and the remission group and that two kinds of samples were obtained from the same subjects (n = 20). The microbiome was analyzed through pyrosequencing of the 16S rRNA gene. From LEfSe results, *Porphyomonas*, *Fusobacterium*, *Treponema*, *Tannerella,* and other periodontal pathogens were abundant in the active group, while lactic acid bacteria (*Lactobacillales* and *Bifidobacterium*) were abundant in the remission group.

## Introduction

Dental implants have a long-term survival rate in many cases^1–3^, and implant-based prosthetic replacements are a widely preferred alternative to conventional fixed or removable prostheses. However, with an increase in the function time of implants, peri-implantitis has become a major concern among 28–56% of the patients and is a leading cause of implant loss^4,5^. Therefore, effective prevention and management of peri-implantitis are essential in maintaining the quality of life and health of the patients. Ailing implants are often managed with antimicrobial, surgical, and local irrigation^6^.

Since the pathogenesis and clinical symptoms of peri-implantitis are similar to that of periodontitis^7^, its management is also based on periodontal therapy. Most patients with periodontitis respond well to treatment, and have stable periodontal tissue in the long term^8^. Similarly, over the treatment course of peri-implantitis, the progression of bone resorption may be arrested and the clinical condition may stabilize. However, in some cases the clinical conditions such as bone resorption, suppuration, and bleeding progress rapidly^9^. The reason for this existing difference is unknown. In addition, peri-implantitis progresses faster than periodontitis in animal models^10^. Several studies have reported a predominance of common microbiota in these two diseases^11,12^. Studies investigating the microbiome associated with peri-implantitis using culture-independent experimental methods, such as deoxyribonucleic acid (DNA) hybridization and 16S rDNA sequencing, have revealed the predominant microbiota unique to peri-implantitis sites and those that are common between peri-implantitis and periodontitis^13–17^. Pyrosequencing of polymerase chain reaction (PCR)-amplified 16S rRNA is an innovative method of arrays that concurrently produce thousands of sequences from individual samples. This novel volume of data enables fully inclusive research of taxonomically distinct communities, revealing the microbial diversity of this disease^18–20^.

However, these reports of bacterial flora analyses do not distinguish between cases of remission and active disease. Thus, it is unclear how the microbiota alters with the changes in disease progression.

In this study, we evaluated the differences in the microbiota associated with remission and active peri-implantitis.

## Methods

### Implants selection and diagnosis

**Figure 1:**
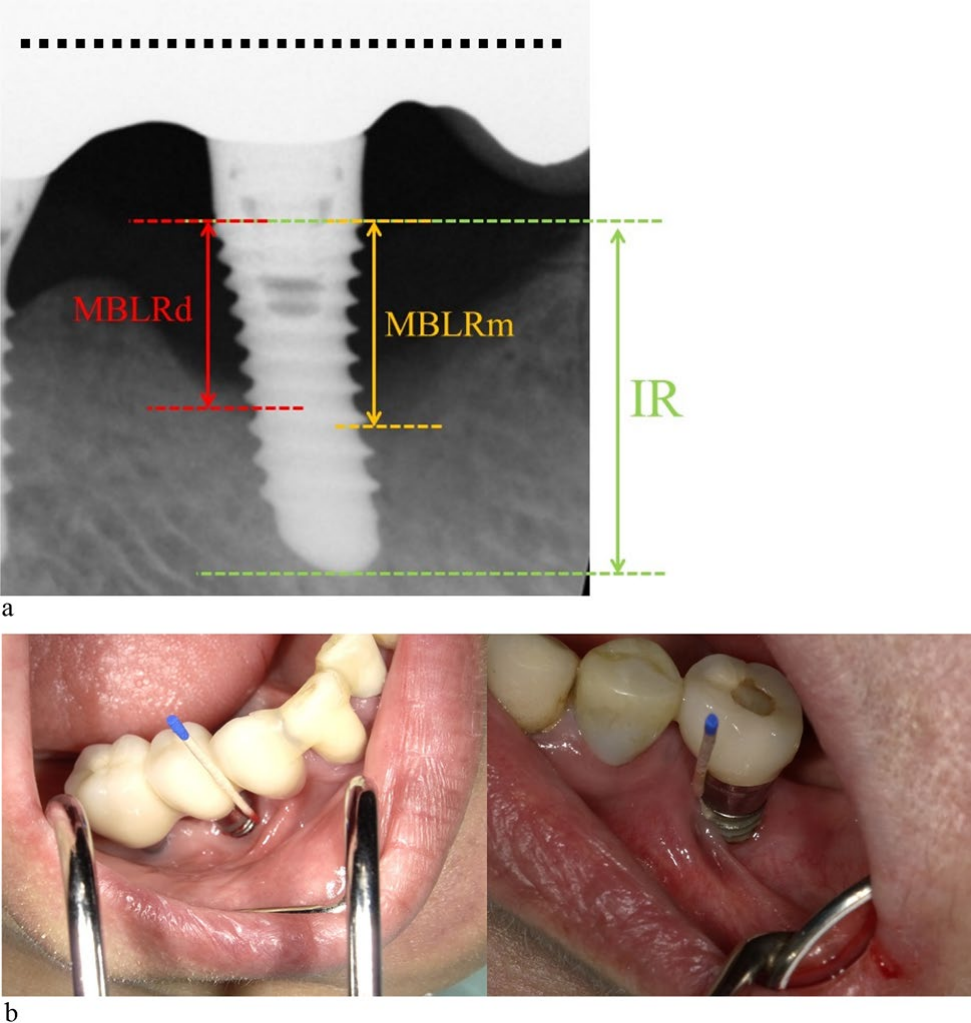
(**a**) Samples were taken from the same oral cavity for both the active and remission peri-implantitis. The extent of bone resorption was calculated by measuring the distance from the implant shoulder (for tissue level, the boundary between the roughened and polished surface) to the most coronal portion of bone-to-implant contact. Implant length of radiograph (IL) is distance from implant shoulder to the most apical site.Marginal bone loss around the implant of radiograph (MBLR) is average distance from the implant shoulder to the most coronal position of bone-to-implant contact in the mesial (MBLRm) and distal (MBLRd) aspects of the implant. The measurement values of marginal bone loss around the implant (MBL) were calculated using the actual implant length (I) and the following ratio: I/MBL = IR/MBLR Peri-implantitis cases with suppuration and bleeding were assigned to the active group, while those without suppuration and bleeding were assigned to the remission group. (**b**)The implant with suppuration is shown.

The diagnostic criteria for peri-implantitis were referred to the 2017 World Workshop Paper^5^. In this study, we defined the remission group as those with stabilized symptoms (suppuration and bleeding) after local irrigation for peri-implantitis, and the active group as those with persistent symptoms. The inclusion criteria for this study were; peri-implantitis diagnosed according to the criteria of the 2017 workshop, presence of both remission and active groups in the same mouth, proper positioning and proper occlusion, at least one year after placement and follow-up of at least six months. The depth of the peri-implant pocket, suppuration and bleeding were confirmed by probing^21,22^,. Bone resorption was confirmed through the radiographic examination^21,22^, furthermore the extent of bone resorption was calculated by measuring the distance from the implant shoulder (for tissue level, the boundary between the roughened and polished surface) to the most coronal portion of the intraosseous part of the implant(Fig. 1a).

### Patients

This study was approved by the Ethics Committee for Epidemiology of Hiroshima University (approval no. 3572) and performed in accordance with the " Ethical Guidelines for Medical Research Involving Human Subjects, Hiroshima University. The exclusion criteria were presence of systemic diseases, antibiotic use, pregnancy, smoking and diabetes. Informed consent was obtained from all participants. A total of eight patients with both two kinds of peri-implantitis were included(Fig. 1b).

### Sample collection

The implants of the patients with peri-implantitis in this study were categorized as active group and remission group. Further, two kinds of samples were obtained from the same subjects (n = 20). Plaque samples from the peri-implant pockets at the greatest probing depth were obtained using a sterile paper point. Paper points were placed into sterile collection tubes and stored at − 80 °C. Following clinical examination and sample collection, the peri-implant tissues were irrigated as per the usual method.

### Metagenomic DNA isolation, 16S rRNA gene library preparation, and sequencing

The paper point sample was transferred to an Eppendorf safe lock tube and processed using a PureLink™ Microbiome DNA Purification Kit (Thermo Fisher Scientific™, Waltham, MA, USA) according to the manufacturer’s protocol. The quantity of DNA was assessed using a spectrophotometer (NanoDrop 2000™, Thermo Fisher Scientific, Waltham, MA, USA).

The v3–v4 hypervariable regions of the bacterial 16S rRNA gene were amplified by PCR with primers (Fw: TCGTCGGCAGCGTCAGATGTGTATAAGAGACAGCCTACGGGNGGCWGCAG, Rv: GTCTCGTGGGCTCGGAGATGTGTATAAGAGACAGGACTACHVGGGTATCTAATCC) and sequenced using an Illumina MiSeq sequencer (Illumina, San Diego, CA).

The process of DNA isolation, gene library preparation, and sequencing was performed at the H. U. Group Research Institute G. K. (Tokyo, Japan). The researcher and bioinformatician were blinded to the clinical information of the patients, except for their group.

### 16S data processing

The raw sequence data were analyzed using the QIIME2^23,24^ (version 2021.4.0). The characterization of amplicon sequence variants (ASV) was processed by DADA2. Singletons and rare ASV (bellow 0.001%) were removed. Taxonomy was assigned to ASV using the q2-feature-classifier based on the SILVA 138.1.

### Statistical analyses

Multivariate tests were performed using Permanova. The Kruskal–Wallis test was applied to compare the differences in alpha diversity of microorganisms (observed features, Simpson index, and Chao-1 index). Bray–Curtis and UniFrac distances were used for the microbial beta diversity analysis. Differences in the relative presence of the microbiome were determined using linear discriminant analysis (LDA) effect size (LEfSe)^25–27^, which can statistically extract features that can explain differences in populations of microbial communities under multiple conditions. The histogram and cladogram were based on LefSe. The non-parametric factorial Kruskal–Wallis sum-rank test was used for detecting features with significant differential abundance with respect to the group. Features violating the null hypothesis were further analyzed, which tested whether all pairwise comparisons between samples in different groups significantly agree with the group level trend using the unpaired Wilcoxon rank-sum test. LEfSe's α parameter for pairwise tests was set to 0.05, and the threshold on the logarithmic score of LDA analysis was set to 2.0. The heat map was plotted with rank-normalized abundances (Kruskal–Wallis and Wilcoxon signed-rank tests, *P* < 0.05).

## Results

### Clinical information

There were eight patients who met the criteria for inclusion in this study, and consent was obtained from all eight patients. There were eight patients who met the criteria for inclusion in this study, and all eight patients gave consent on each clinic day at the outpatient department. Number of implants per pt is 6.0 per patent. Number of sites designated active and remission per patient is 1.6/2.1 per patient. Nine cases with suppuration and bleeding and 11 cases without suppuration and bleeding were observed in eight patients. Bone resorption and gingival erythema were observed around all the implants. The demographic and clinical characteristics of the patients are presented in Table 1.

**Table 1:** Clinical information of patients.

A								
Patients	A	B	C	D	E	F	G	H
Active								
sample No	1	2	3	4	5	6, 7	8	9
Remission sample No	10	11	12	13	14	15, 16, 17	18	19, 20

B								
	Active group (sample No.1–9)	Remission group (sample No.10–20)						
Age (years)	69.25 ± 9.64							
Sex (% females)	100	100						
Probing depth (mm)	5.9 ± 1.7	4.6 ± 1.8						
Retention type (Screw/Cement)	7/2	9/2						
Suppuration (+ / −)	9/0	0/11						
Bleeding on probing (+ /−)	9/0	0/11						
Bone resorption (mm)	7.6 ± 3.0	4.3 ± 3.8						
Level of the abutment connection (bone/tissue)	8/1	10/1						
Implant brand (Brånemark/Straumann)	7/2	9/2						

### Microbial diversity of peri-implantitis

**Figure 2:**
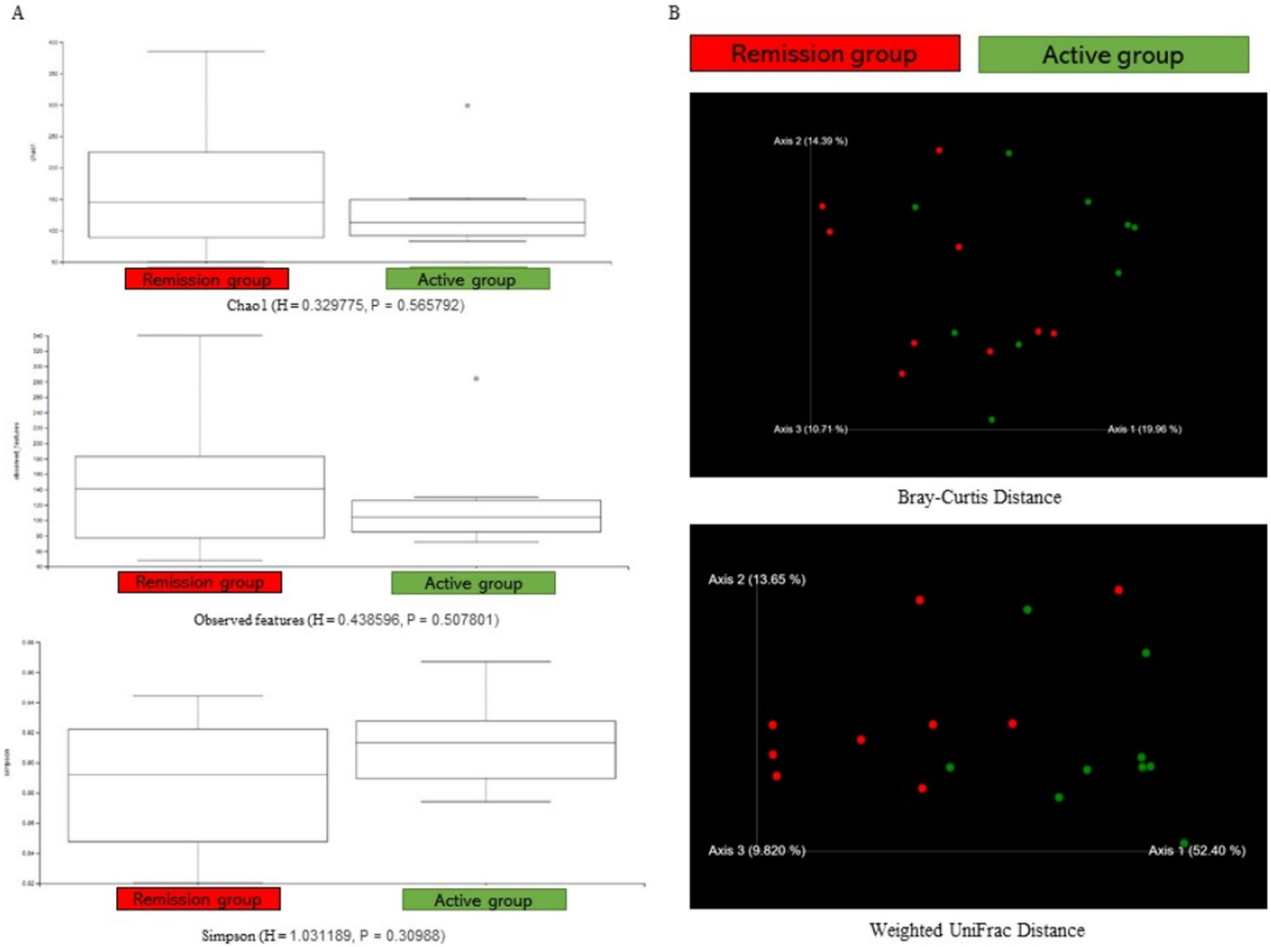
The oral microbiota difference between the active group (n = 9) and the remission group (n = 9). (**a**) Calculation of alpha diversity values (Chao1, observed features, and Simpson) for comparison of the total microbial diversity of peri-implantitis between the active group (n = 9) and the remission group (n = 9). (**b**) The principal coordinate analysis charts based on Bray–Curtis and weighted UniFrac distance: comparisons between the two groups.

Two samples (Nos. 12 and 17) had insufficient DNA extraction or insufficient amplification by PCR. Next-generation sequencing was used for the detection, and the number of reads was the same as that of the negative control sample. The differences in the microbial diversity and composition between the active and remission groups were examined (Fig. 2). There was no significant difference between the two groups in any of the indices. However, the active group had a higher Simpson index and a lower Chao1 index and observed features than the remission group. The trend was different because the Simpson index gave weightage to major species, whereas the Chao1 index gave weightage to rare species. According to the differences in trends among the indices, the active and remission groups had different microbiomes. The remission group had several rare species, while the active group had an even diversity of species. In the principal coordinates analysis, the active and remission groups formed different clusters in the ray curve distance and weighted UniFrac distance, suggesting a microbial shift from the original microbiome consisting of rare species with low pathogenicity to the inflammation-inducing microbiome, as in other inflammatory diseases of the oral cavity.

### Taxa classification

**Figure 3:**
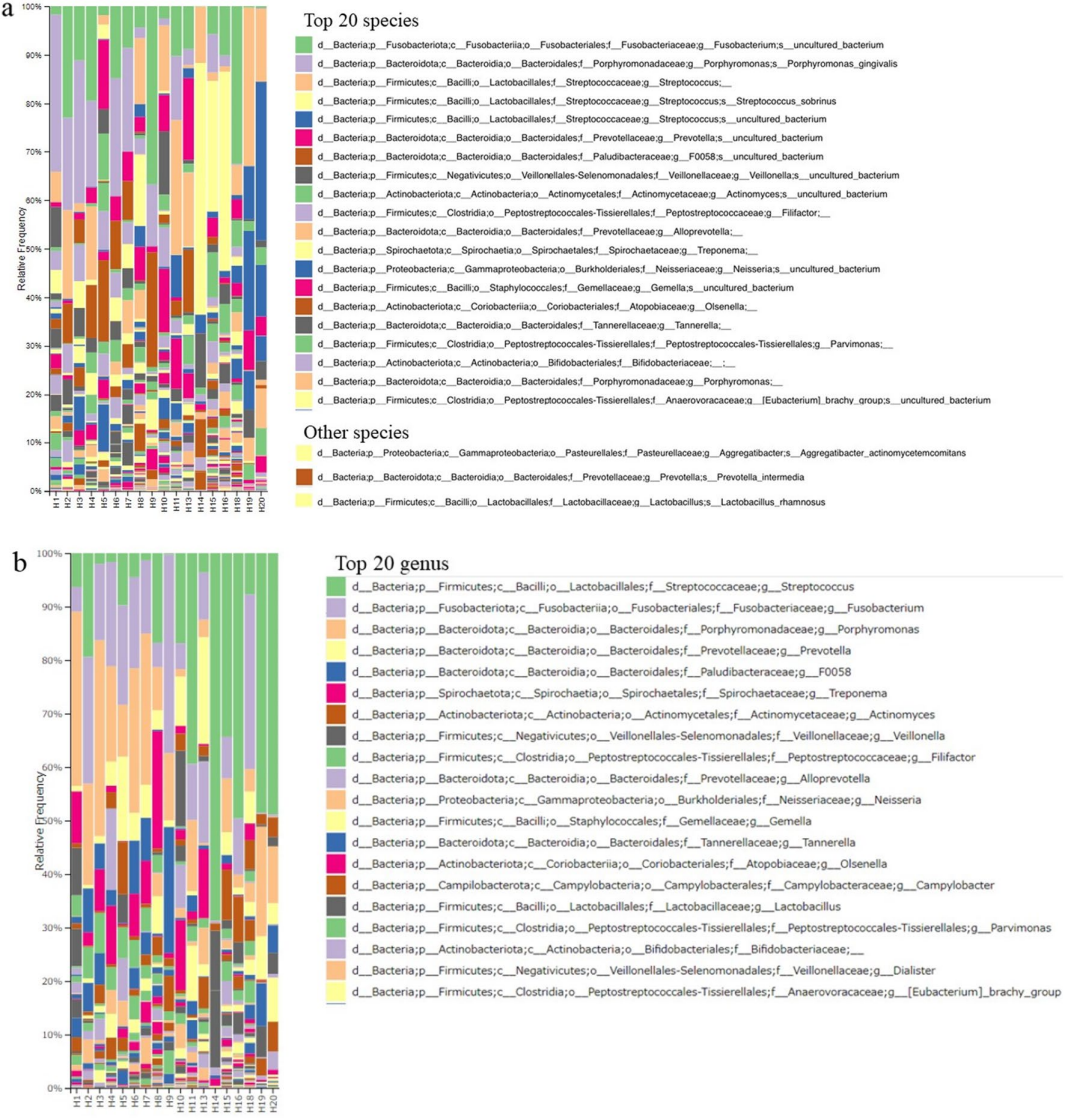
The taxa bar plot shows that the relative frequency of bacterial compositions. The three most dominant taxa of peri-implantitis are *Fusobacterium* (uncultured bacterium), *Porphyromonas gingivalis*, and genus *Streptococcus. Treponema denticola*, *Prevotella intermedia*, and *Aggregatibacter actinomycetemcomitans* are the bacteria associated with periodontal disease other than *Porphyromonas gingivalis*. Lactic acid bacteria, including *Lactobacillus* and *Bifidobacterium*, are rare taxa. Moreover, *Lactobacillus rhamnosus*, *Lactobacillus fermentum*, and *Enterococcus faecalis* which are utilized as probiotics, are detected.

**Figure 4:**
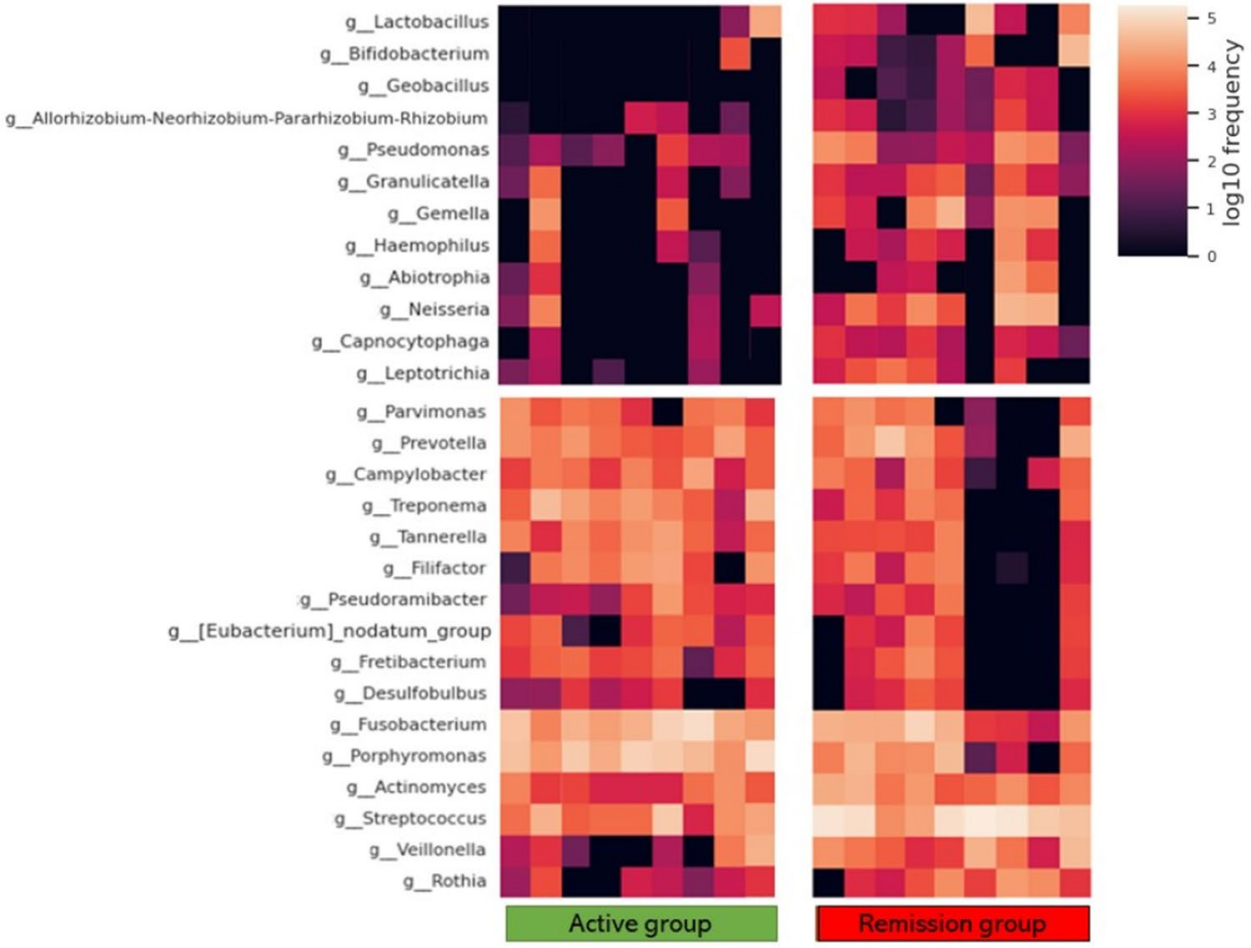
Heat map of relative abundance of the major bacteria in the two groups by taxa. *Fusobacterium*, *Porphyromonas*, and *Streptococcus* show high abundance and taxa bar plot. *Lactobacillus* and *Bifidobacterium* tend be more abundant in the remission group than in the active group.

The taxa bar plot shows the relative frequency of the bacteria. The three most dominant bacteria of peri-implantitis were *Fusobacterium* (uncultured bacterium) species, *Porphyromonas gingivalis*, and *Streptococcus* species*.* Bacteria associated with periodontal disease other than *P. gingivalis*, such as *Treponema denticola*, *Prevotella intermedia*, and *Aggregatibacter actinomycetemcomitans* were detected. Lactic acid bacteria, including *Lactobacillus* and *Bifidobacterium*, were rare. In addition, *Lactobacillus rhamnosus*, *Lactobacillus fermentum*, and *Enterococcus faecalis*, which are utilized as probiotics, were detected. The other dominant taxa are shown in Fig. 3a*. Streptococcus*, *Fusobacterium*, *Porphyromonas*, *Prevotella*, *Treponema*, *Actinomyces*, *Veillonella*, *Filifactor*, *Alloprevotella*, *Neisseria*, *Gemella*, *Tannerella*, *Olsenella*, *Campylobacter*, *Lactobacillus*, *Parvimonas*, *Dialister*, and *Eubacterium* brachy groups were the most frequent at the genus level (Fig. 3b). The taxa heat map showed the differences in the relative abundances between the samples (Fig. 4). The results of the phylogenetic tree for each sample were similar for the same subjects. The abundance of *Fusobacterium*, *Porphyromonas*, and *Streptococcus* can be observed in the taxa bar plot. *Lactobacillus* and *Bifidobacterium* were more abundant in the remission group than in the active group.

### Compositional differences between the groups

**Figure 5:**
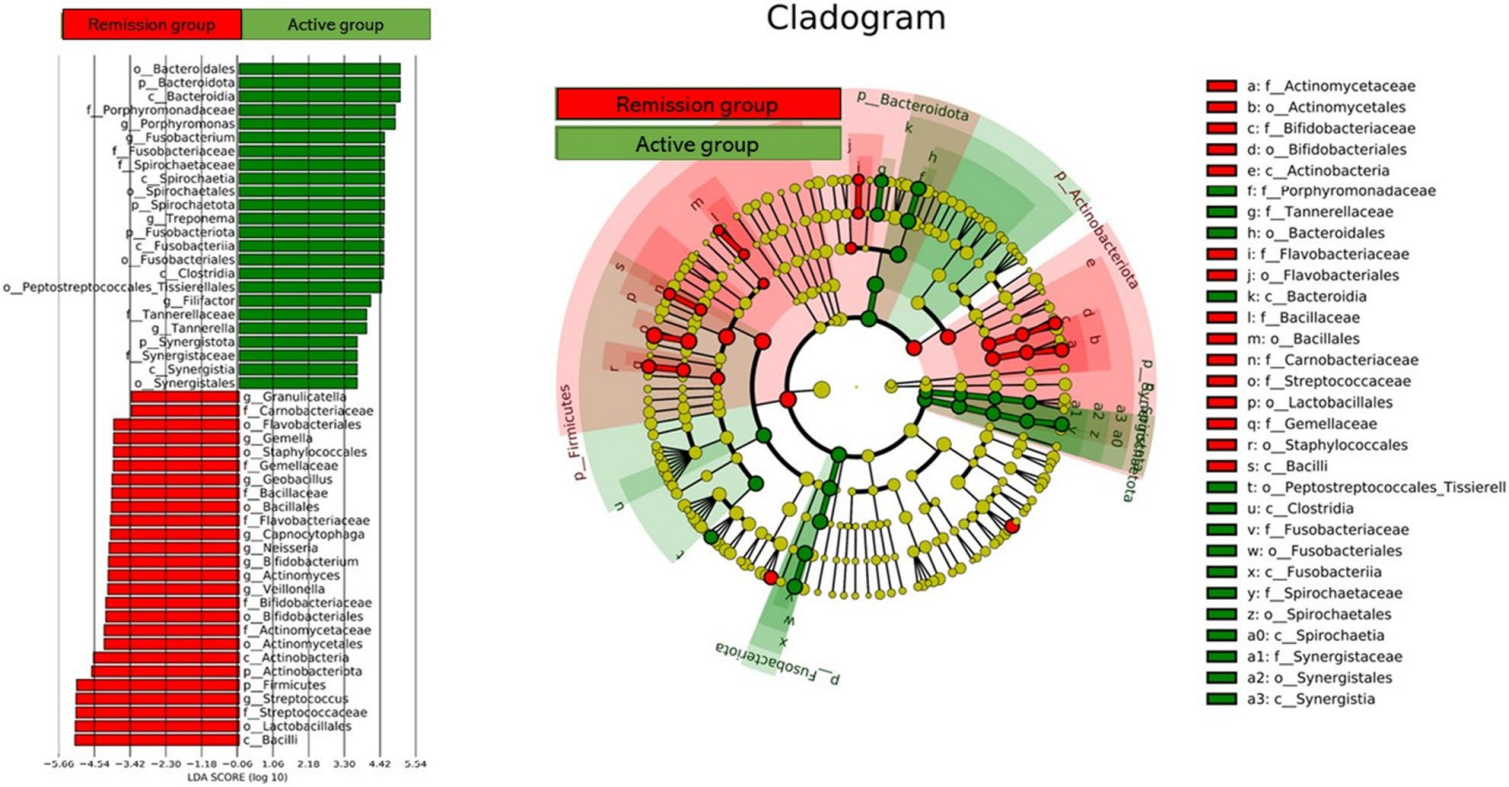
g_*Porphyomonas*, g_*Fusobacterium*, g_*Treponema*, and g_*Tannerella* are more abundant in the active group, whereas o_*Lactobacillales* and g_*Bifidobacterium* are more abundant in the remission group.

LEfSe analysis was performed to compare the relative abundance (differential abundance between the groups with LDA score > 2 and *P* < 0.05). g_*Porphyomonas*, g_*Fusobacterium*, g_*Treponema*, and g_*Tannerella* were more abundant in the active group, while o_*Lactobacillales* and g_*Bifidobacterium* were more abundant in the remission group. Bacteria considered as the causative pathogens of peri-implantitis were more common in the active group, while bacteria that were used as probiotics^28–31^ were more common in the remission group. Other LDA scores and cladograms are shown in Fig. 5.

## Discussion

This study describes an association between suppuration at implant sites and a trend towards a change in bacterial spp. composition relative composition of spp. within the sampled biofilm. Although the generalization of this sampling result (100% females, aged approx 60–80) is unclear, the strength of this study is that all patients were sampled at both active and remission sites. This sampling method help account for individual variations. Furthermore, we successfully identified some representative bacteria at the species level. Several reports compare the microbiomes of healthy implant sites with those of peri-implantitis or peri-implant mucositis^16,17^ or the microbiomes of periodontitis with those of peri-implantitis^14,15^ and reports of bacterial clusters associated with peri-implantitis also exist^32^. These results of diversity and phylogenic of microbiota differed widely among studies because the definition of peri-implantitis is ambiguous and because clinical conditions vary. In this study, there was no significant difference in alpha diversity, but the active group tended to have a higher Simpson index and a lower Chao1 index and observed features. Active inflammation may increase the relative abundance of certain flora and decrease the relative abundance of minor flora. Alpha diversity is the measure of the diversity within a sample. In other words, it is a sample-specific index that reflects a greater diversity of species when the values are high. The importance of “the number of different species observed” or “the equal observation of each species” differs depending on the index. Principal coordinate analysis using beta diversity showed that they formed different clusters, indicating that the samples from the remission and active groups show different trends within each group, although the flora composition was similar within the groups. Beta diversity is the measure of the difference in the diversity between two samples and is expressed as the distance between two points. The greater the distance, the greater the difference in the composition of both samples.

The relative frequency of periodontal disease-causing bacteria, such as *P. gingivalis*, was higher in the active group on the taxa bar plot, while the relative presence of lactic acid bacteria was lower in the active group on the taxa heat map. The “keystone pathogen”^33^ and “microbial shift” hypotheses^34^ suggest that the number of malignant bacteria can increase due to the nutrient supply to the biofilm, causing the biofilm to become more pathogenic. In the present study, *P. gingivalis* showed a higher relative amount in the active group, which is consistent with these hypotheses. Thus, changes in the peri-implant environment lead to an association between the tissue and microbiota from symbiosis to dysbiosis. The presence of common bacterial species in the active and remission groups and the fact that their compositional ratios are subject to microbial shifts due to environmental factors may reflect these theories. These results indicate that peri-implant inflammation is associated with changes in the bacterial flora structure. In the taxa bar plot of this study, the major bacterial taxa of peri-implant sites were partially similar to that of periodontitis. In particular, the high prevalence of *P. gingivalis* in the active group far exceeded our expectations.

Periodontal bacteria, such as *P. gingivalis*, *T. denticola*, *Tannerella forsythia*, *A. actinomycetemcomitans*, *P. intermedia*, *Fusobacterium*, *Campylobacter*, and other periodontal pathogens, have been reported to be associated with implant disease. *P. gingivalis*, *T. denticola*, *A. actinomycetemcomitans*, *P. intermedia*, *Fusobacterium*, and *Campylobacter* were also annotated in the samples used in this study. *Porphyromonas*, *Fusobacterium*, *Treponema*, and *Tannerella* were more prevalent in the active group, as indicated by the LDA score. Thus, these bacteria may be the keystone species in the bacterial flora.

The level of inflammation and pocket depth should be considered when an ailing implant is identified during SPIT. If inflammation is intractable, additional treatments such as flap surgery and probiotic therapy should be considered.

Some of the lactic acid bacteria, utilized as probiotics^30,31,35,36^, identified in this study in the remission group, such as *L. Rhamnosus and Bifidobacterium*.

These findings suggest that lactic acid bacteria may have an effect on implantitis activity by periodontal pathogens. However we evaluated only the differences in the microbiota associated with remission and active peri-implantitis and not focused on lactic acid bacteria. Thus it is unclear how the microbiota alters with the changes in disease progression. The clinical study in the future should consider patients on probiotic therapy in the exclusion criteria, the possibility of confounding cannot be ruled out and aim to explore this aspect further in our future studies.
